# Oxyresveratrol Modulates Genes Associated with Apoptosis, Cell Cycle Control and DNA Repair in MCF-7 Cells

**DOI:** 10.3389/fphar.2021.694562

**Published:** 2021-07-01

**Authors:** Sarayut Radapong, Kelvin Chan, Satyajit D. Sarker, Kenneth J. Ritchie

**Affiliations:** ^1^ Toxicology Laboratory, Medicinal Plant Research Institute, Department of Medical Sciences, Ministry of Public Health, Nonthaburi, Thailand; ^2^ Centre for Natural Products Discovery, School of Pharmacy and Biomolecular Sciences, Liverpool John Moores University, Liverpool, United Kingdom

**Keywords:** oxyresveratrol, artocarpus lakoocha, anticancer, gene expression, microarray

## Abstract

Oxyresveratrol (OXY) is a small molecule phytochemical which has been reported to have important biological function. The aim of this study was to elucidate the gene expression and biological pathways altered in MCF-7, breast cancer cells following exposure to OXY. The cytotoxicity to different cancer cell lines was screened using MTT assay and then whole gene expression was elucidated using microarray. The pathways selected were also validated by quantitative PCR analysis, fluorometric and western blot assay. A total of 686 genes were found to have altered mRNA expression levels of two-fold or more in the 50 μM OXY-treated group, while 2,338 genes were differentially expressed in the 100 µM-treated group. The relevant visualized global expression patterns of genes and pathways were generated. Apoptosis was activated through mitochondria-lost membrane potential, caspase-3 expression and chromatin condensation without DNA damage. G0/G1 and S phases of the cell cycle control were inhibited dose-dependently by the compound. Rad51 gene (DNA repair pathway) was significantly down-regulated (*p* < 0.0001). These results indicate that OXY moderates key genes and pathways in MCF-7 cells and that it could be developed as a chemotherapy or chemo-sensitizing agent.

## Introduction

**FIGURE 1:**
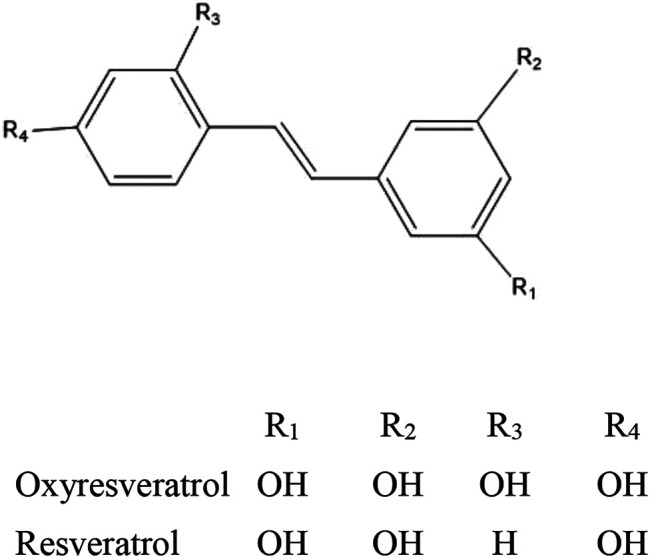
Chemical structure of OXY and resveratrol.

Oxyresveratrol (OXY) (C_14_H_12_O_4_, M.W. 244.24 g/mol) is a naturally occurring polyphenol found to be particularly concentrated in the heartwood of *Artocarpus lakoocha* Wall. ex Roxb (family: Moraceae), an indigenous plant in Thailand (Radapong et al., 2020). The compound is in the group of small molecule hydroxystilbenoids such as resveratrol, pterostilbene, gnetol and piceatannol, which have been reported to possess various potent bioactivities such as cardioprotection, neuroprotection, anti-diabetic properties, depigmentation, anti-inflammation, cancer prevention and treatment (Akinwumi et al., 2018). However, several biological activities are unique to OXY (antivirus and antihelminthics) (Galindo et al., 2011; Preyavichyapugdee et al., 2016). In consideration of its chemical structure shown in Figure 1, which is similar to the well-known antioxidant, resveratrol, it was hypothesized that it may also share similar anticancer properties to resveratrol. Previously Chatsumpun et al. (2016) reported that OXY exhibited cytotoxicity to breast, cervical and lung cancer cell lines. However, the precise mechanism involved in the modulation of carcinogenesis remains to be elucidated.

**FIGURE 2:**
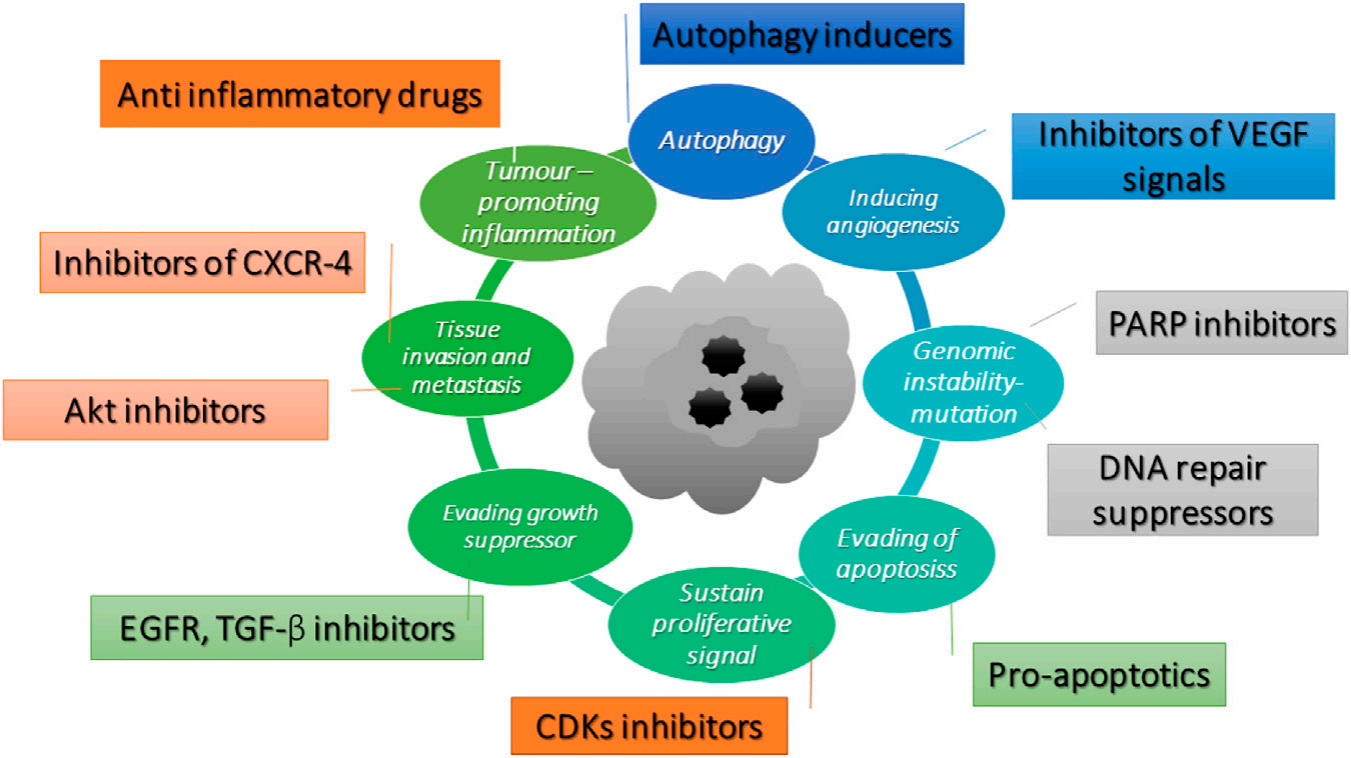
Potential molecular targets of anti-cancer compounds adapted from Hanahan and Weinberg (2011).

The cancer hallmarks which identify cancer targets have been illustrated by Hanahan and Weinberg (2011). The hallmarks involve defects in the regulation of cell proliferation and homeostasis, which include evading apoptosis, self–sufficiency in growth signals, evading growth suppressor, tissue invasion and metastasis, promoting inflammatory, autophagy, inducing angiogenesis and genomic instability. The potential molecular targets of an anticancer compound are illustrated in Figure 2. Pro-apoptotics and apoptosis inducers that target the apoptosis pathway are an effective way to treat to all types of cancer (Pfeffer and Singh, 2018). OXY has been reported to induce the intrinsic pathway of apoptosis in neuroblastoma cells (Rahman et al., 2017). Rahman et al. (2017) have provided supporting evidence of the apoptotic cell morphology changing and that OXY–treated cells shrank and became rounded with membrane blebbing and apoptotic vacuoles. The molecular targets of this pathway included BAX and BCL–2 proteins, which triggered the cascade proteins through mitochondrial apoptosis. Several phytochemicals inhibit cancer cell proliferation by modulating the genes that control several aspects of the cell cycle. Cyclin–dependent kinases (CDKs) and E3 ubiquitin ligases mainly play a crucial role throughout the process (Duronio and Xiong, 2013). Resveratrol is found to cause growth inhibition of human epidermoid carcinoma (A431) cells via cell cycle arrest. Treatment with resveratrol (1–50 μM for 24 h) causes an induction of WAF1/p21 that inhibits cyclin D1/D2–CDK6, cyclin D1/D2– CDK4, and cyclin E–CDK2 complexes, thereby imposing an artificial checkpoint at the G1→S transition of the cell cycle (Ahmad et al., 2001). OXY suppressed cell migration of Jurkat T cells in response to stromal cell-derived factor 1 (SDF–1). The mechanistic study indicated that OXY diminished CXCR4 –mediated T–cell migration via inhibition of the MEK/ERK signalling cascade (Chen et al., 2013). OXY has been reported to exhibit autophagy independently from apoptosis in neuroblastoma cells. The molecular targets were mostly *via* inhibition of PI3K/AKT/mTOR/pS6 signalling and activation of p38 MAPK pathway (Rahman et al., 2017). OXY also showed anti-inflammatory properties in murine macrophage cell line RAW 264.7 by inhibiting the nitric oxide synthase (iNOS) and cyclooxygenase-2 (COX-2) expression through down-regulation of NF-kB binding activity (Chung et al., 2003). Poly (ADP–ribose) polymerases (PARPs) are enzymes involved in DNA–damage repair. Recently, inhibition of PARP has emerged as a promising strategy for targeting cancers with defective DNA–damage repair, (Livraghi and Garber, 2015). The original concept of the activity of PARP inhibitors was that they acted through synthetic lethality by targeting the base excision repair pathway (BER) in BRCA–deficient tumours. Therefore, disruption of the two pathways led to cell death (Iglehart and Silver, 2009). Resveratrol has been reported to show cleavage induction of PARP1 (Demoulin et al., 2015). The compound also reduced the expression of genes in homologous recombination (HR) of the DNA damage repair pathway including RAD recombinase 51 (RAD 51) in MCF-7 cells, enhancing the antiproliferative effect of cisplatin (Leon-Galicia et al., 2018). RAD51 is an important part of the mechanism and has been observed that the gene was overexpressed in chemoresistant cancers ((Slupianek et al., 2001; Hannay et al., 2007). Therefore, elucidation the of the mechanism of action of OXY using a high throughput tool such as microarray assay would allow greater understanding of how cells respond to OXY.

This study aims to investigate the biological response to OXY in MCF-7 cells focusing on cytotoxic and anti-cancer effects by elucidating key genes and biological pathways involved.

## Materials and Methods

### Cell Lines and Reagents

Human caucasian lung carcinoma cells (A–549), human colorectal cancer cells (CACO–2), human caucasian hepatocyte carcinoma cells (HepG2), human breast cancer cells (MCF-7), Caucasian prostate adenocarcinoma cells (PC–3), Mouse macrophage cells (RAW 264.7) and the two non-transformed cell lines including Human foetal lung cells (MRC–5) and normal human breast cells (MCF10A) were from the European collection of authenticated cell cultures (ECACC) (Salisbury, United Kingdom). A–549, CACO–2, HepG2, MCF-7, and MRC–5 cells were maintained in Eagle’s minimum essential medium (EMEM); RAW 264.7 cells were cultured in Dulbecco’s modified eagle’s medium (DMEM); PC–3 were in RPMI 1640 medium from Thermo Fisher Scientific (New York, NY, United States) supplemented with 10% fetal bovine serum in a 5% CO_2_ incubator at 37°C, while MCF10A cells were maintained in DMEM/F12 medium supplemented with 5% horse serum, 20 ng/ml epidermal growth factor, 0.5 mg/ml hydrocortisone, 100 ng/ml cholera toxin, 10 μg/ml insulin and 1x pen/strep. OXY (>97%) was obtained from Sigma-Aldrich (Buchs, Switzerland). The stock solution was freshly prepared by dissolving in dimethyl sulfoxide (DMSO) and diluted in the culture medium.

### MTT Proliferation Assay

The cells were plated at a density of 7 × 10^3^ cells per well in a 96-well plate to determine the cytotoxic effect of OXY. Cells were incubated for 24 h and then treated with 1.56–200 µM OXY for 48 h. After incubation, 0.5 mg/ml MTT [3-(4,5-Dimethylthiazol-2-yl)-2,5-Diphenyltetrazolium Bromide] was added to each well and incubated for 4 h followed by 200 µL DMSO. Absorbance was measured at 570 nm using Clariostar microplate reader (MBG Labtech GmBH, Ortenberg, Germany). The data were normalized to the untreated (control) cells at 100% viability. The 50% inhibitory concentration (IC_50_) was calculated using non-linear regression (curve fit) analysis, GraphPad software and Doxorubicin was used as a standard drug.

### Gene Expression Profiling

MCF-7 cells were seeded at 2.5 × 10^5^ cells/well in 6-well plates for 24 h, then they were treated with OXY at the concentrations of 0, 50, and 100 μM for 24 h. Total RNA of each sample was extracted using the RNeasy Mini Kit (Qiagen, Hilden, Germany) pre-treated with on-column DNA digestion with an RNase-free DNase kit (Qiagen, Hilden, Germany). According to the manufacturer’s protocol, RNA was re-suspended in 30 μL of nuclease-free water and stored at −80°C until further analysis. The amount and quality of the RNA samples were analyzed by NanoDrop 2000 (Thermo Fisher Scientific, New York, NY, United States). The samples were run on an Agilent RNA 6,000 Pico chip to assess RNA integrity using the 2,100 Expert Software RNA 6,000 Pico kit through 2,100 Bioanalyzer instrument, which was proceeded by the 2,100 Expert Software (Agilent Technologies, Palo Alto, CA, United States).

Total RNA was processed using GeneChipTM WT PLUS Reagents (Thermo Fisher Scientific, Palo Alto, CA, United States) containing WT amplification kit module1, WT amplification kit module2, Poly-A RNA Control Kit, WT Terminal Labeling Kit and Hybridization Control Kit. The samples were hybridized in biological triplicate to the Human Clariom S arrays (a 400 format array) (Affymetrix Inc., Santa Clara, CA, United States), following the manufacturer’s recommendations.

Briefly, 100 ng of the pooled RNA was converted into the first-strand cDNA. Second-strand cDNA synthesis was followed by an *in-vitro* transcription to generate cRNA. The cRNA products were used as templates for the second cycle of cDNA synthesis, where deoxyuridine triphosphates were incorporated into the new strand. The cDNA was then fragmented using a uracil-DNA glycosylase and apurinic/apirymidinic endonuclease. The fragments (50–70 mers) were then labelled by means of a biotin-labelled deoxynucleotide terminal addition reaction. The labelled cDNA product was heated to 99°C and hybridized to each array for 16 h at 45°C. Samples were washed with stain cocktail and the array-holding buffer on the GeneChipTM Fluidics Station 450 (Thermo Fisher Scientific, Palo Alto, CA, United States).

A GeneChip 3000G scanner (Affymetrix Inc.) and the Expression Console software (Affymetrix Inc.) were used to obtain fluorescent signals and quality control data of the scanned arrays. Signal intensities from each array were analyzed using Partek Genomic Suite version 6.4 (Partek, St Louis, MO, United States).

The microarray data were deposited at the NCBI GEO database (GSE151139). The Transcriptome Analysis Console (TAC) Software (Thermo Fisher Scientific, Palo Alto, CA, United States) was used for the visualization of the expression data in the biological pathways context. The data set was analyzed using this tool and a gene expression fold change > 2 or < −2. Ebayes Anova was used for statistical analysis with a *p*-value < 0.05.

### Real-Time Reverse-Transcription Polymerase Chain Reaction

cDNA was synthesed using QuantiTect Rev. Transcription Kit (Qiagen, Valencia, CA, United States) following the manufacturer’s protocol. Briefly, 2-µg template RNA was added to the reverse-transcription master mix and then the samples tubes were incubated at 42°C for 15 min. The cDNA samples were tested in triplicate with quantitative PCR using a QuantiTect SYBR Green PCR Reagents kit (Qiagen, Valencia, CA, United States). Total 2 µL of each sample was mixed with SYBR Green PCR Master Mix and 10x QuantiTect Primers (Qiagen, Valencia, CA, United States). Real-time (RT-PCR) was then performed following the manufacturer’s protocol in a Rotor-Gene Q (Qiagen, Valencia, CA, United States). mRNA ratios relative to the Glyceraldehyde 3-phosphate dehydrogenase (GAPDH) housekeeping gene were calculated for the standardization of gene expression levels. A melting curve analysis was also performed to verify the specificity and identity of PCR products. Finally, the products were run on agarose gels to check the specificity of the PCR.

For selected genes, the data were analyzed using the equation described by Livak and Schmittgen (Livak and Schmittgen, 2001) as follows: the amount of target = 2^−ΔΔCt^. The average Δ_Ct_ from OXY untreated MCF-7 cells as a calibrator for each gene tested. Data were presented as mean ± SD.

### Quantification of Apoptotic Cells

Apoptosis was detected with the PE Annexin V apoptosis detection kit I (BD Biosciences Inc, San Jose, CA, United States). MCF-7 cells were seeded in 6–well plates and then treated with OXY at different concentrations of 0, 25, 50 and 100 μM for 0, 3, 6, and 24 h. The cells were trypsinized and washed twice with ice–cold phosphate-buffered saline (PBS), and then cells were re–suspended at a concentration of 1 × 10^6^/ml cells in binding buffer. A total of 100 μL of the cell suspension was transferred into a 2 ml micro centrifuge tube to which 5 μL PE annexin V and 5 μL 7–amino–actinomycin D (a vital nucleic acid dye) were added. The cells were gently mixed and incubated in the dark for 15 min at room temperature. Finally, a 400 μL of binding buffer was then added to each tube and the apoptotic cells were quantified using the flow cytometer (BD Biosciences, San Jose, CA) within 1 h. Cells that stained positive for PE annexin V and negative for 7–amino–actinomycin D were undergoing apoptosis; cells that stain positive for both PE annexin V and 7–amino–actinomycin D were either in the end stage of apoptosis, undergoing necrosis, or were already dead; and cells that stain negative for both PE annexin V and 7–amino–actinomycin D were alive and not undergoing measurable apoptosis.

### Determination of Caspase–3 Expression Induced by Oxyresveratrol

Active caspase–3 staining protocol was performed following manufacturer’s instructions (PE active caspase–3 apoptosis kit, BD biosciences, San diego, CA, United States). Total 5 × 10^5^ cells of MCF-7 treated with different concentrations of OXY were harvested and transferred into 15 ml centrifuge tubes and then the suspension was centrifuged at 300 × g for 5 min at 4°C. Cells were washed twice with 1 ml ice–cold PBS and then re–suspended in 0.50 ml BD Cytofix/Cytoperm™ solution. The cells were incubated on ice for 20 min. After that the buffer was removed, the cells were washed twice with 0.50 ml BD Perm/Wash™ buffer at room temperature. Then, the cells were re–suspended with 100 μL BD Perm/Wash™ buffer plus antibody and incubated for 30 min at room temperature. Finally, the cells were washed with 1 ml washing buffer and re–suspended in 0.50 ml washing buffer. The cells were maintained at 4°C until analysed by flow cytometer.

### Mitochondrial Membrane Potential (Δ*Ψ*m) Analysis by JC–1 Fluorescence

Cellular mitochondrial dysfunction can be reflected by the loss of the mitochondrial membrane potential, which can be indirectly measured by the fluorescent probe JC–1 using BD™ MitoScreen Kit (BD Biosciences, Sandiego, CA, United States). The protocol was followed according to the manufacturer’s instruction. Briefly, MCF-7 cells treated with different concentrations of OXY were harvested and transferred into 15 ml centrifuge tubes and then the suspension was centrifuged at 300 × g for 5 min at 4°C. Then the cells were re–suspended with 0.50 ml JC–1 working solution and incubated at 37°C in a cell’s incubator for 15 min. The cell pellet was washed twice with 1X assay buffer (2 and 1 ml, respectively). Finally, the cells were re–suspended with 0.50 ml 1X assay buffer, then analysed by flow cytometry.

### Nuclear Morphological Detection Using Hoechst 33,342 Staining

The nuclear morphological changes of chromatin condensation and chromosome fragmentation induced by OXY were examined using hoechst 33,342 the staining was carried-out according to the manufacture’s instructions. Briefly, the cells were treated with different concentrations of OXY in 6–well plates for 24 h. The 10 mg/ml Hoechst stock solution was prepared and diluted with PBS (1:2000). The medium was removed from each well and then 500 μL of the dye working solution was added. The plate was incubated in the cell incubator for 10 min. Afterwards, the cells were washed with 1 ml PBS for three times. The cells were viewed under fluorescence microscope (Olympus, Hamburg, Germany). The mode of cell death was then determined in terms of distinct morphological changes, including membrane blebbing, nuclear and cytosolic condensation and nuclear fragmentation in the treated group compared to the untreated cells which served as the control.

### Measurement of DNA Damage by Comet Assay

The comet assay was performed under alkaline conditions. Cells were seeded in 6–well tissue–culture plates. They were treated with 50 and 100 µM OXY. After 24 h of exposure with OXY or 100 µM H_2_O_2_ for 4 h as the positive control, cells were collected by trypsinization, washed with PBS and resuspended in ice–cold PBS. 10 μL of the resuspended cells was mixed with 100 μL of low melting point agarose at 37°C and spread the suspension over the well with the pipette tip. The slides were placed at 4°C in the dark until gelling occurred and then immersed in pre–chilled lysis buffer at 4°C. After 60 min incubation, the buffer was aspirated and replaced with pre–chilled alkaline solution for 30 min at 4°C. After lysis and unwinding, the slides were placed in a horizontal electrophoresis tank filled with freshly prepared alkaline electrophoresis buffer. The electrophoresis was run for 25 min at 15 V and 300 mA. After electrophoresis, the slides were transferred to pre–chilled distilled water and immersed for 2 min, aspirated and repeated twice. The final water rinse was aspirated and replaced with cold 70% ethanol for 5 min. Thereafter, the slides were allowed to air dry and 100 μL/well of diluted Vista Green DNA dye was added to each slide for 15 min in the dark at room temperature for DNA staining. DNA migration was observed using fluorescence microscope at a magnification of 10X (Leica Microsystems CMS GmbH, Germany).

### Cell Cycle Analysis Using Flow Cytometry

Cell cycle analysis was conducted by BD Cycletest™ Plus kit (BD biosciences, San Diego, CA, United States) using propidium iodide as a DNA stain to determine DNA content in the cells. The effect of OXY treatment on the cell cycle was determined by flow cytometry as described by the manufacturer’s instruction. Briefly, MCF-7 cells were seeded at 2.5 × 10^5^ cells/well in 6–well plates. MCF-7 cells were treated with OXY at the concentration of 25, 50, and 100 μM for 3, 6 and 24 h. Cells were trypsinized with 300 μL of 0.25% trypsin solution for 3 min in the cell incubator. Total 200 μL of fetal bovine serum was added and then all of the cell suspension was transferred into a 15 ml centrifuge tube. The suspension was centrifuged at 300 rcf for 5 min. The supernatant was discarded, the cell pellet was re–suspended with 1 ml buffer solution (contains sodium citrate, sucrose, and DMSO). The cells were centrifuged and re–suspended one more time and then counted using a hemacytometer for 5 × 10^5^ cells. Cells were frozen in the freezer (–80°C) for PI staining. The cell suspensions were thawed in water bath, centrifuged at 400 g for 5 min at room temperature (20–25°C). All the supernatant was carefully decanted. A 250 μL of solution A (trypsin buffer) was added to each tube and gently mixed by tapping the tube, incubated for 10 min at room temperature. Afterwards, a 200 μL of solution B (trypsin inhibitor and RNase buffer) was added to each tube and gently mixed by tapping the tube by hand, and incubating for 10 min at room temperature. Finally, a 200 μL solution C (PI stain solution) was added to each tube and incubated for 10 min in the dark on ice or in the refrigerator (2–8 °C) until analyzed by flow cytometer. A total number of 1 × 10^4^ cells was subjected to cell cycle analysis using the flow cytometer.

### Western Blot

Cells were seeded at 2.5 × 10^5^ cells/well on 6-well dishes and allowed to adhere overnight. Cells were then treated with OXY at the concentrations of 50 and 100 µM for 24 h and then washed twice with ice-cold PBS. The cells were then lyzed in lysis buffer (protease inhibitor cocktail) left on ice for 5 min then, transferred to centrifuge tubes, sonicated for 30 s with 50% pulse and then centrifuged at ∼14,000 ×g for 15 min to pellet the cell debris. The supernatants were then collected, and protein concentrations determined by Bradford assay (Bio-Rad, Richmond CA, United States). An equal amount of protein sample (20 μg) was resolved by a volume of 2x Laemmli sample buffer, then boiled for 5 min 7.5% Mini-PROTEAN^®^ TGX Stain-Free™ protein gels (Bio-Rad, Richmond, CA, United States) were used and run at 180 V for 1 h. Protein transfer was achieved using Trans-Blot^®^ Turbo™ mini PVDF transfer packs (Bio-Rad, Richmond, CA, United States). Proteins were transferred onto PVDF membrane at 1.3 A for 7 min. Membranes were then blocked with 5% skim milk/TBST for 1 h and then probed with the indicated primary antibody overnight at 4°C and then blotted with appropriate horseradish peroxidase-conjugated secondary anti-rabbit antibody. Visualization was performed using an enhanced chemiluminescence kit (BioRad Inc., Hercules, CA, United States) with Clarity™ Western ECL Substrate. Protein level was normalized to the matching densitometric value of the internal control *β*-actin.

### Statistical Analysis

Results of MTT assays were expressed as mean ± standard error. Significant differences in genes in WikiPathways were detected using Fisher’s Exact Test. Statistical significance was assessed by One-way analysis of variance followed by Dunnett’s test or the unpaired *t*-test using GraphPad Prism 9 (GraphPad Software, La Jolla, CA, United States).

## Results

### MTT Cytotoxicity

MTT assay was used to analyze the viability of the cells treated with different concentrations of the compound for 48 h. The total DMSO concentration in the cell culture medium did not exceed 0.5% which was found not to affect the cell growth or viability of the cells. After 48 h of treatment, OXY significantly inhibited cell growth and viability in a dose-dependent manner to MCF-7, HepG2, PC–3, RAW 264.7 and A–549 cells with the IC_50_ values of 30.64 ± 4.79, 104.47 ± 0.82, 109.35 ± 9.63, 115.95 ± 11.28, and 148.63 ± 4.48 µM, respectively. Whereas, the compound showed no toxicity to CACO–2, MCF10A and MRC–5 (IC_50_ > 200 µM). IC_50_ values of OXY and doxorubicin are shown in Table 1.

**TABLE 1:** Inhibitory concentration (IC_50_, µM) of OXY to the cells.

	Oxyresveratrol (µM)	Doxorubicin (µg/ml)
A–549	148.63 ± 4.48	0.54 ± 0.02
CACO–2	>200	3.27 ± 1.06
HepG2	104.47 ± 0.82	0.84 ± 0.08
MCF–7	30.64 ± 4.79	0.21 ± 0.07
PC–3	106.90 ± 8.63	3.08 ± 0.48
RAW 264.7	115.95 ± 11.28	0.21 ± 0.07
MRC–5	>200	0.37 ± 0.01
MCF10A	>200	NA

### Effect of Oxyresveratrol on the Human Breast Cancer Cells Gene Expression Profiles

**FIGURE 3:**
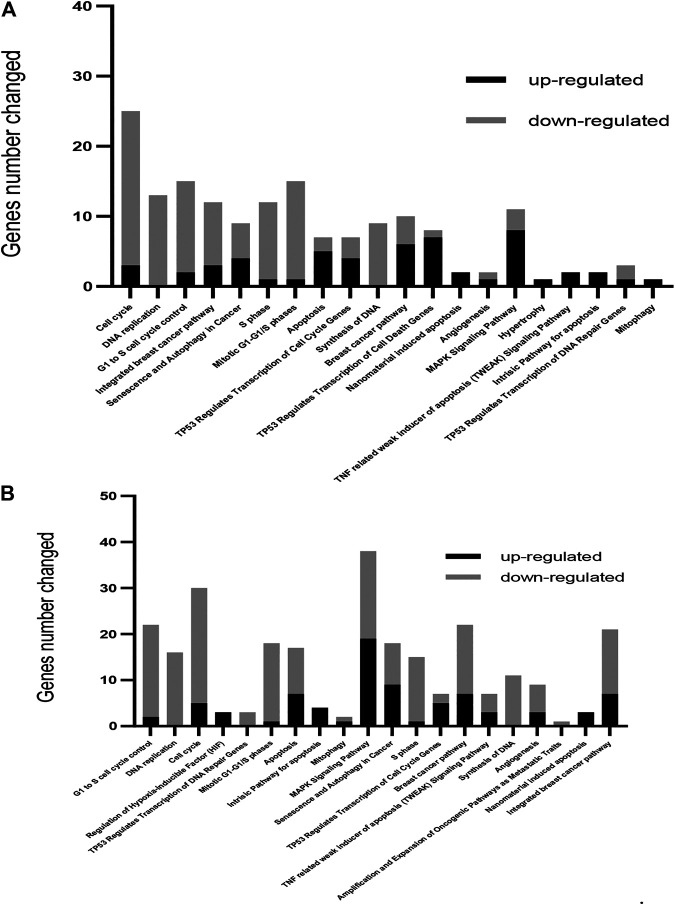
Gene ontology-based biological process pathways altered by OXY in MCF-7 breast cancer cells. Differentially expressed genes in MCF-7 cells treated with 50 µM **(A)** or 100 µM **(B)** of OXY for 24 h. To determine the biological process and pathways involved, the list of significantly upregulated and downregulated genes was analyzed using Fisher’s Exact Test, and then analyzed using WikiPathways.org.

Global expression patterns of genes and pathways were obtained using microarray technology and data analysis software. Gene expression changes occurring as a consequence of the exposure of MCF-7 cells to 50 or 100 µM OXY for 24 h was performed as described in the materials and methods section. The number of differentially expressed genes under both conditions compared to the control group were observed. The cellular pathways in which these differentially expressed are found were shown in Figure 3 *.*

A total of 686 genes were differentially expressed in the 50 µM-treatment group; with 262 genes being upregulated and 424 downregulated. Total 2,338 genes were differentially expressed in the 100 µM-treated groups; among these, 907 were upregulated and 1,431 were downregulated (showed in the Supplementary Materials). WikiPathways (wikipathways.org), a biology community maintained website that displays biological pathways, was utilized to identify pathways and the functions of genes altered by OXY treatment in MCF-7 cells.

From this analysis, it was found that the first 20 most affected pathways (arranged from significant values in the 100 μM-OXY treatment) following OXY treatment were associated with cell cycle control, apoptosis and DNA repair, as well as the senescence and autophagy processes. It was also noted that the majority of the genes in cell cycle control and DNA repair were down regulated, while those in apoptosis and autophagy were up regulated.

**FIGURE 4:**
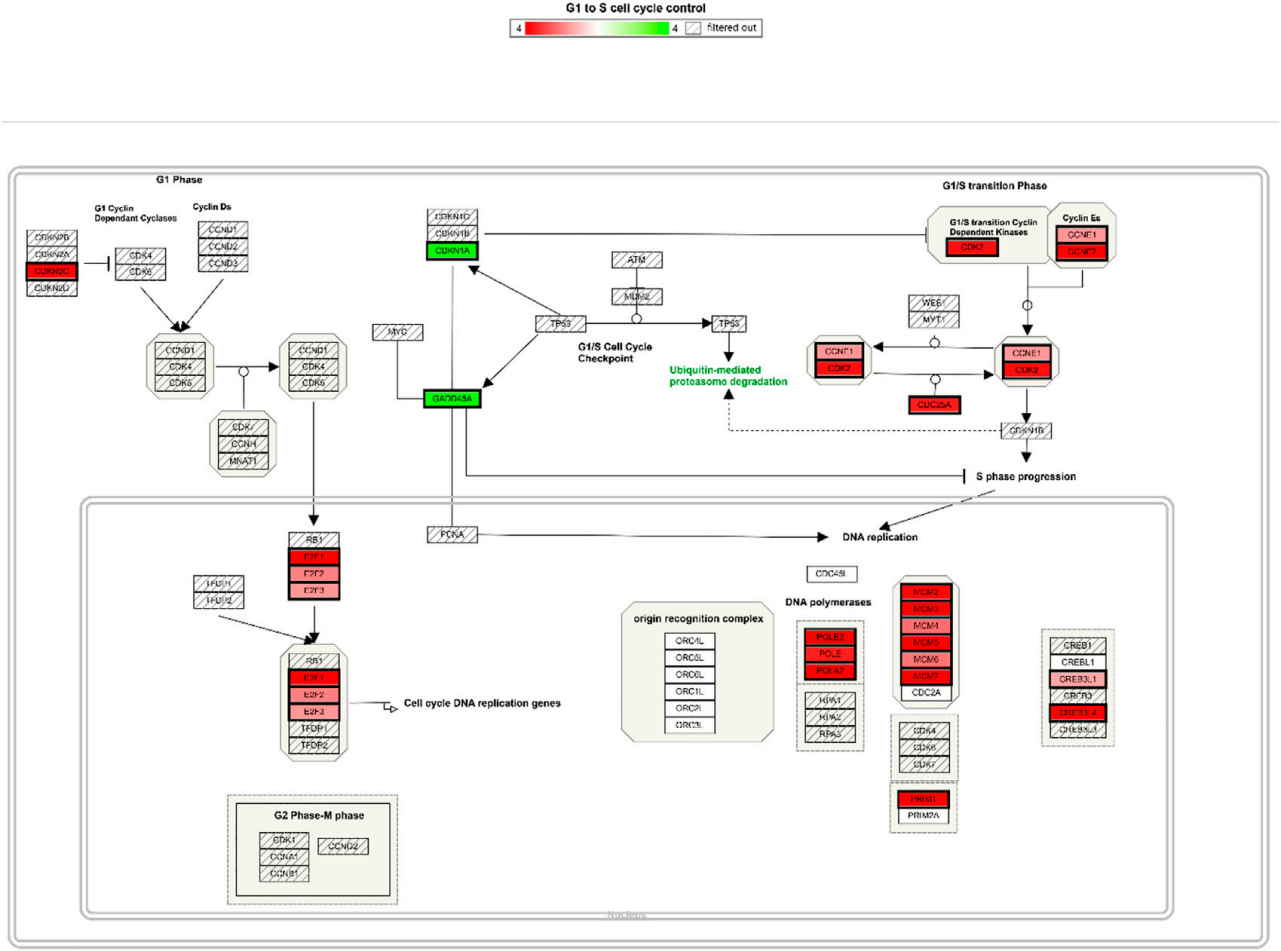
Cell cycle control pathway integrating expression data for MCF-7 cells treated with 100µM OXY for 24 h. Genes labelled in green are up regulated and in red are down regulated.

Among these, the most dramatically affected pathways were that of cell cycle control. At both 50 and 100 μM OXY, the expression of DNA-damage-inducible, alpha (*GADD45A*) and cyclin-dependent kinase inhibitor 1A (*p21*, *CDKN1A*) were up regulated. Whereas, cyclin-dependent kinase 2 (*CDK2*), E2F transcription factor one (*E2F1*) were down regulated (Figure 4).

### Validation of Differentially Expressed Genes

**FIGURE 5:**
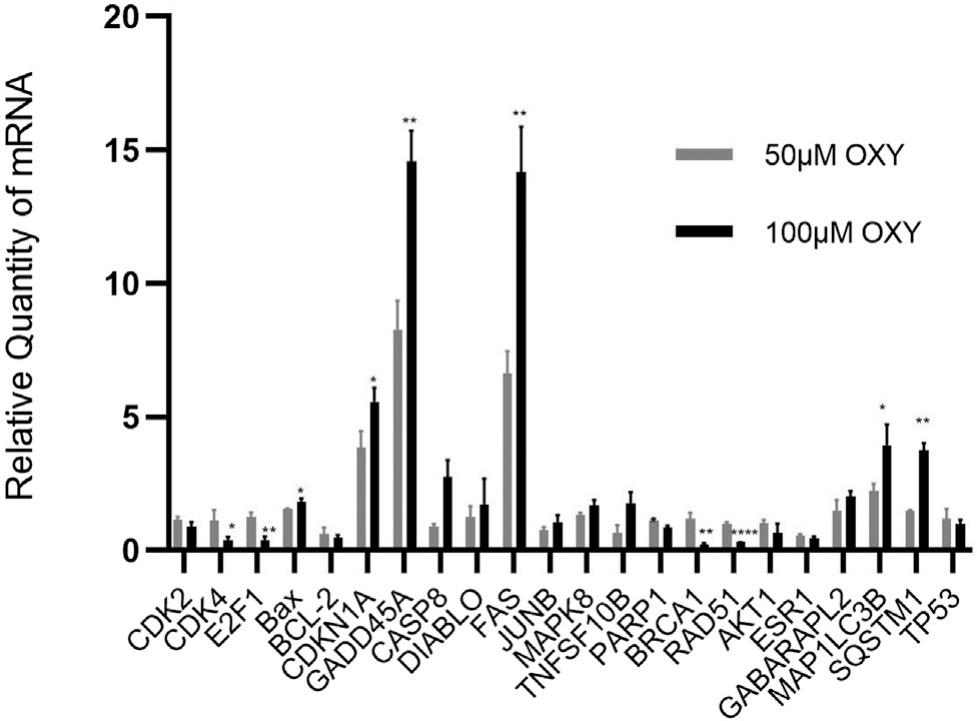
Relative quantity of mRNA expression from MCF-7 cells treated with OXY. Validation of selected genes differentially expressed in MCF-7 cells treated for 24 h with 50 or 100 µM of OXY vs. control data set. Statistical analysis was carried out using unpaired *t* test (*p < 0.5, **p < 0.05 and ****p < 0.0001).

To validate the microarray results, the mRNA expression of selected genes involved in the regulation of cell cycle, apoptosis, DNA repair and autophagy (*CDK2*, *CDK4*, *BCL-2*, *E2F1*, *BAX*, *CDKN1A*, *GADD45A*, *CASP8*, *DIABLO*, *FAS*, *JUNB*, *MAPK8*, *TNFRSF10B*, *PARP1*, *BRCA1*, *RAD51*, *AKT1*, *ESR1*, *GABARAPL2*, *MAP1LC3B*, *SQSTM*, and *TP53*) was confirmed using RT-qPCR. Triplicates were performed for each gene. In all cases, the RT-qPCR data confirmed those obtained by 50 and 100 μM-OXY treated array analyses (Figure 5). The genes involving cell cycle control, DNA replication, apoptosis, senescence and autophagy in cancer pathways including *CDK4*, *E2F1*, *Bax*, *CDKN1A*, *GADD45A*, *FAS*, *GABARAPL2*, and *SQSTM* were significantly upregulated. Whereas, the genes in DNA repair pathway including *PARP1*, *BRCA1*, and *RAD51* were significantly downregulated compared between the two doses treated.

### Quantification of Apoptosis

**FIGURE 6:**
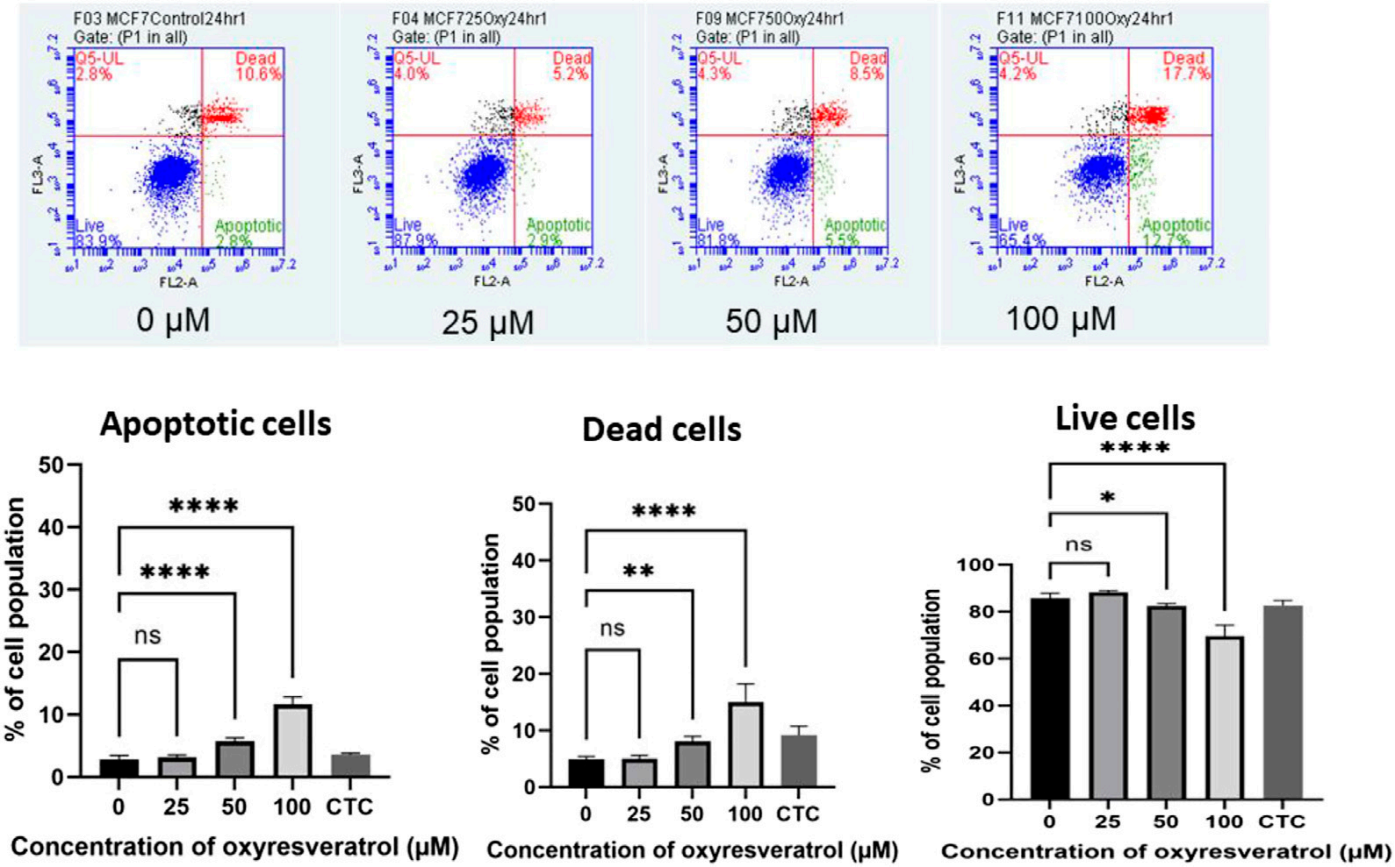
Induction of apoptosis by OXY in MCF-7 cells determined using flow cytometry. Bar graphs showing the percentage of MCF-7 cells in apoptosis, live cells and dead cells when treated with 0, 25, 50 and 100 μM OXY for 24 h and camptothecin (CTC) for 4 h and then subject to flow cytometric analysis. Data are the mean ± SEM of three independent experiments. Statistical analysis was carried out using One-way ANOVA analysis of variance followed by Dunnett’s test. The significant difference was compared relatively to control (*p = 0.037, **p = 0.003 and ****p < 0.0001).

Apoptosis induction by OXY in the cells was determined. The apoptotic cells expressing PS on their cell membranes were detected using Annexin V conjugated with PE. OXY induced apoptosis in MCF-7 cells dose–dependently at the concentrations tested of 25, 50 and 100 µM compared to the untreated control (0 µM) exhibiting the PE Annexin V fluorescence percentage of 3.14 ± 0.37, 5.74 ± 0.53 and 11.69 ± 1.15%, respectively. Following 24 hours of treatment; the cells treated with OXY 25 µM became apoptotic, while those treated with OXY 50 and 100 µM were significantly different from the controls. These results illustrate that most of the cells treated with 50 and 100 µM OXY within 24 h underwent apoptosis and as a consequence died (Figure 6).

### Caspase–3 Expression

**FIGURE 7:**
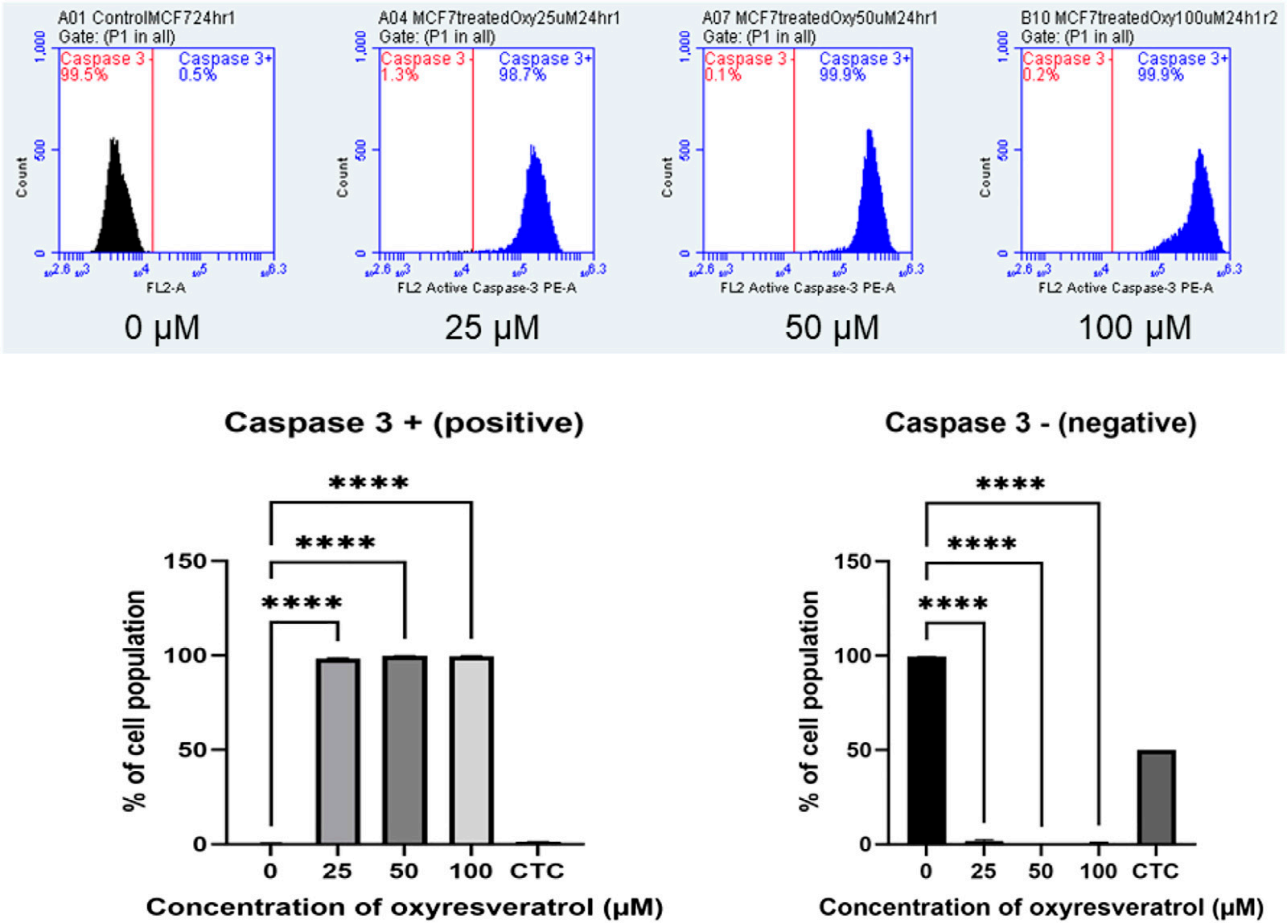
Caspase–3 expression in MCF-7 cells following exposure to various concentrations of OXY for 24 h and camptothecin (CTC) for 4 h. The cells were stained with anti–active caspase–3 and analyzed by flow cytometry. Data are the mean ± SD of three experiments. Statistical analysis was carried out using One-way ANOVA analysis of variance followed by Dunnett’s test. The significant difference was compared relatively to the control (****p < 0.0001).

The apoptotic executioner protein, caspase 3, which made the cells cytomorphological charateristic change was also determined using anti-active caspase–3 conjugated with PE. MCF-7 cells treated with different concentrations of OXY at doses of 25, 50 and 100 µM for 24 h showed a dramatically changed amount of caspase–3 expression on cell membranes compared to the control; more than 98% of the population was caspase–3 positive in all doses treated, while the cell population of the caspase–3 negative decreased proportionally. All treatments were significantly different compared with the control (Figure 7).

### Mitochondrial Membrane Potential (Δ*Ψ*m)

**FIGURE 8:**
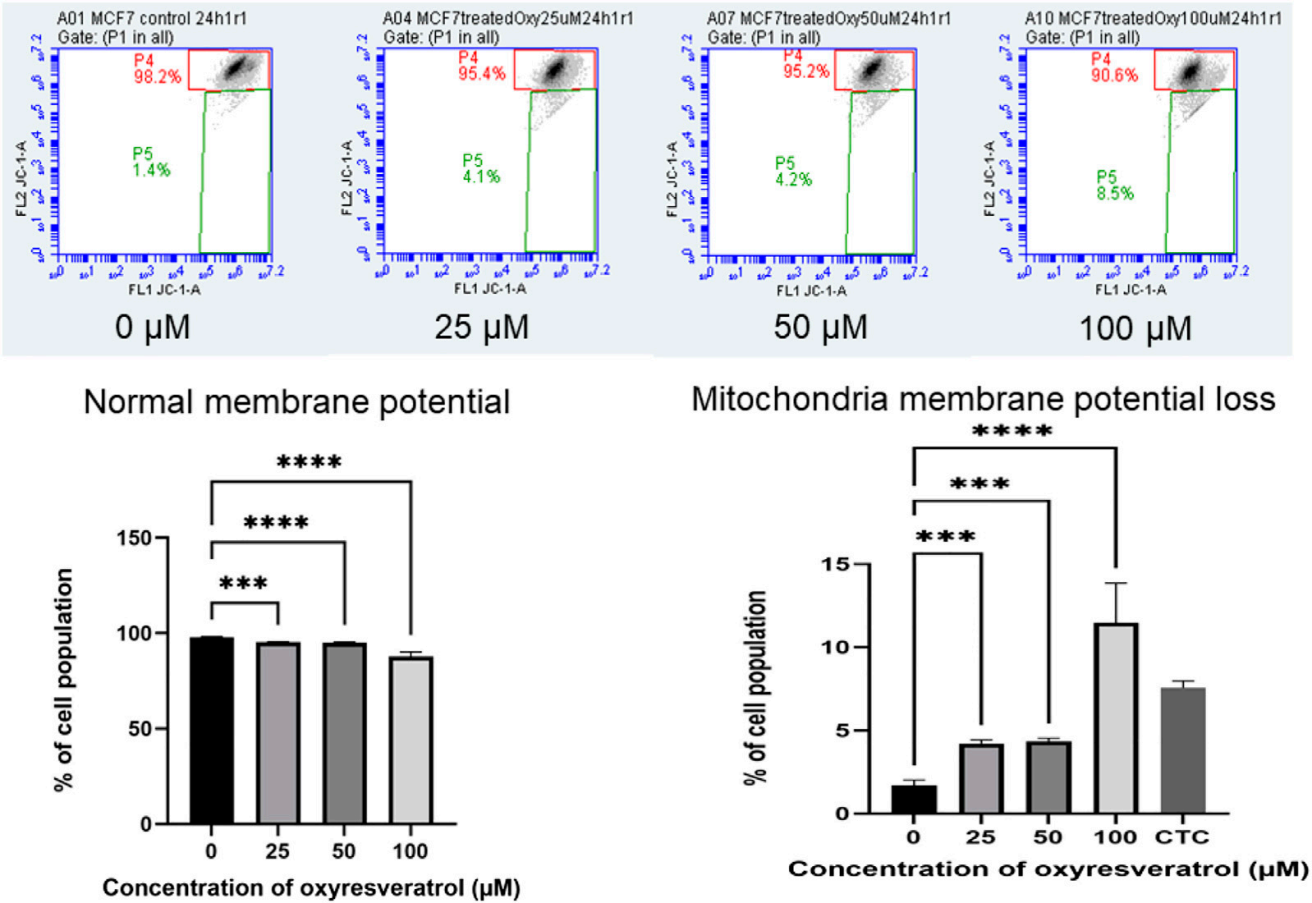
Mitochondrial membrane potential of MCF-7 cells following exposure to various concentrations of OXY for 24 h and camptothecin (CTC) for 4 h. The cells were stained with JC–1 and analyzed by flow cytometry. Data are the mean ± SD of three experiments. Statistical analysis was carried out using One-way ANOVA analysis of variance followed by Dunnett’s test. The significant difference was compared relatively to control (***p < 0.001 and ****p < 0.0001).

Mitochondrial dysfunction is associated with changes in ΔΨm. Mitochondrial membrane depolarization was measured by JC-1. At high ΔΨm, JC-1 the cationic lipophilic probe spontaneously formed J-aggregates with red fluorescence. On the other hand, at low ΔΨm, depolarized mitochondria, JC-1 stayed monomeric resulting in low or no fluorescence signal. As the concentration of OXY increased, the mean values of FL2 FL1−1A gating P4 decreased (normal membrane potential). These results indicate that the mitochondrial membrane was depolarized in a dose-dependent manner. MCF-7 cells lost mitochondria membrane potential dose–dependently following exposure to several concentrations of OXY, which caused the cells to undergo apoptosis. 24–h treatment was found to cause membrane potential loss to a significant degree compared to the untreated control; 4.15 ± 0.08, 4.77 ± 2.56 and 11.47 ± 2.75% for 25, 50 and 100 µM–OXY, respectively (Figure 8).

### Nuclear Morphological Detection

**FIGURE 9:**
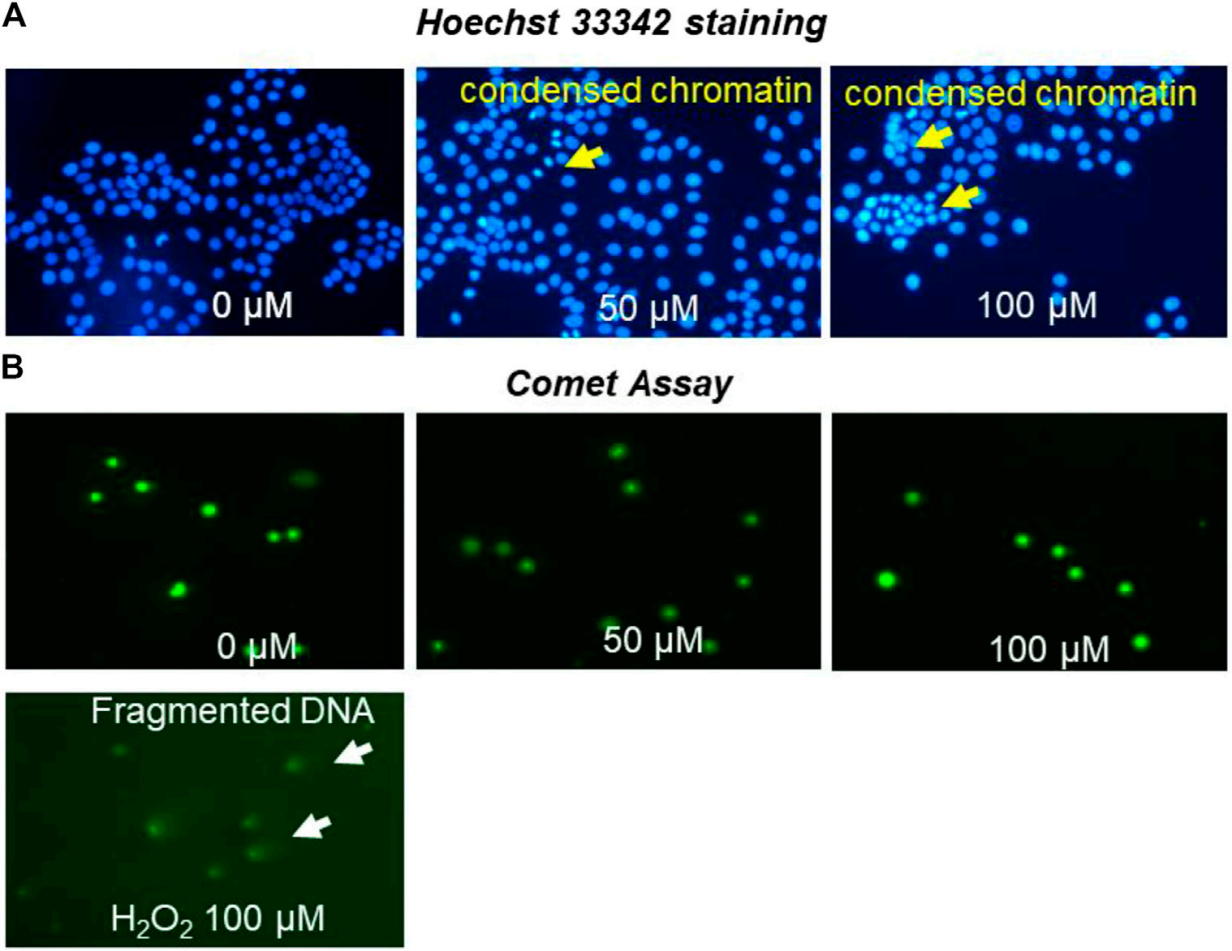
MCF-7 nuclei staining by Hoechst 33,342 **(A)** photomicrographs of stained DNA of MCF-7 cells (alkaline comet assay) **(B)** apoptosis detection following treatment with different concentrations of OXY for 24 h and 100 µM H_2_O_2_ for 4 h.

MCF-7 cells were investigated for nuclear and chromatin changes following treatment with OXY. The pictures of chromatin changed are shown in Figure 9A. MCF-7 cells were observed to have undergone chromatin condensation at all doses used.

### DNA Damage by Comet Assay

MCF-7 cells treated with OXY were investigated for DNA damage as shown in Figure 9B. The cells treated with 50 and 100 µM of OXY for 24 h did not display DNA damage. OXY at the effective doses did not cause damage to DNA in MCF-7 cells.

### Cell Cycle Analysis

**FIGURE 10:**
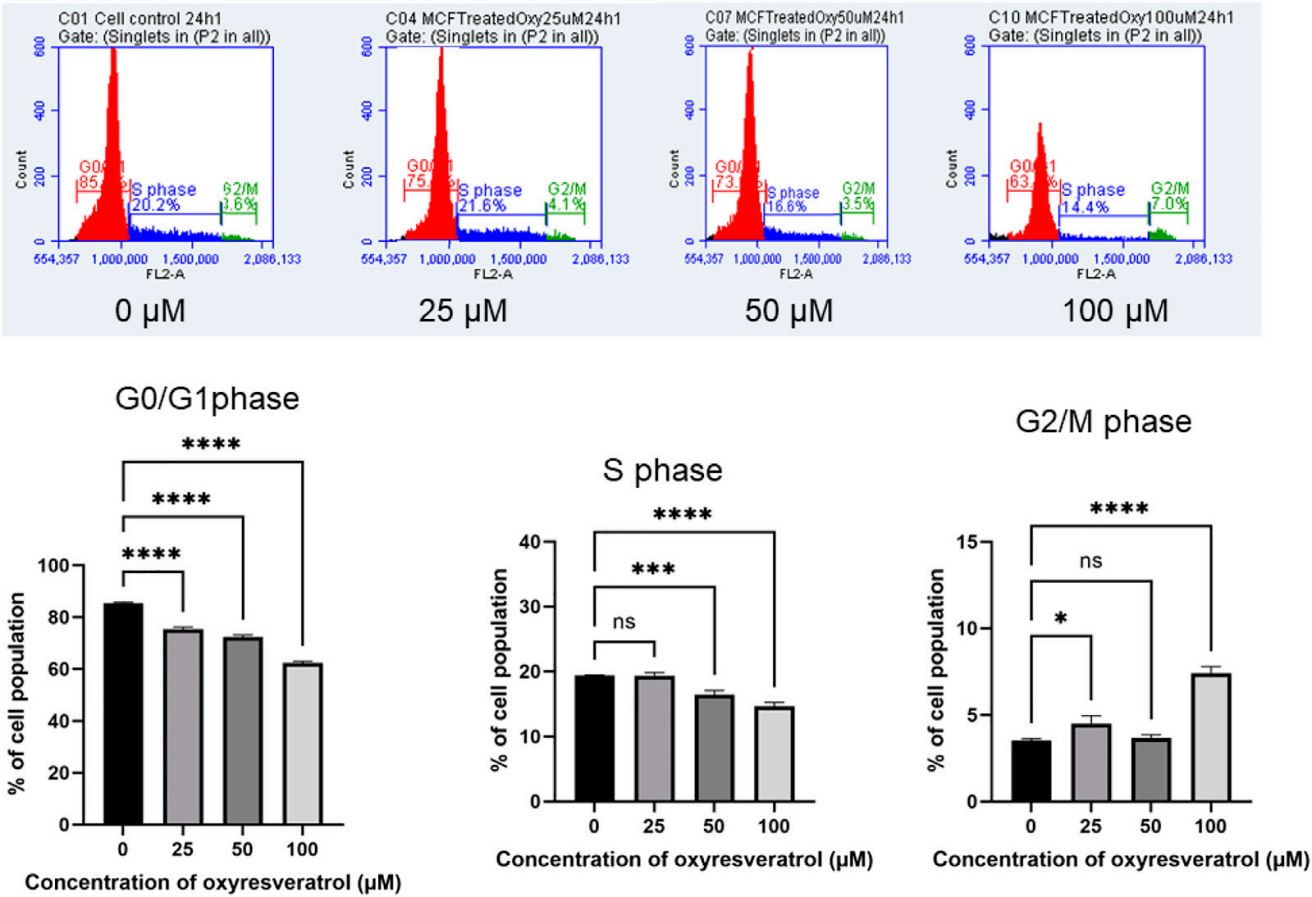
Effect of OXY on cell cycle distribution in MCF-7 cells determined using flow cytometry. The histograms showing the numbers of cells in different phases after MCF-7 cells were treated with 25–100 μM OXY for 3–24 h and then subjected to flow cytometric analysis. Data are the mean ± SD of three experiments. Statistical analysis was carried out using One-way ANOVA analysis of variance followed by Dunnett's test. The significant difference was compared relatively to control (*p = 0.0134, ***p = 0.002 and ****p < 0.0001).

The cell cycle arrest was also analyzed by treating the cells with different doses of OXY. The results in Figure 10 show that the compound decreased DNA content dose-dependently in G0/G1 phase of the cell cycle at 24 h. Compared with the untreated control (0 µM), 25, 50 and 100 µM OXY decreased the percentage of the cell population from 85.43 ± 0.34 to 75.39 ± 0.75, 72.34 ± 0.82, and 62.27 ± 0.68%, respectively. S phase also significantly decreased from 19.37 ± 0.09 to 19.35 ± 0.48, 16.47 ± 0.63, and 14.66 ± 0.57%, respectively. whereas G2/M phase was induced from 3.52 ± 0.11 to 4.50 ± 0.46, 3.68 ± 0.17, and 7.40 ± 0.38%, respectively during the time incubated. These results indicate that OXY arrested G0/G1 and S phases of the cell cycle.

### Western Blot Analysis

**FIGURE 11:**
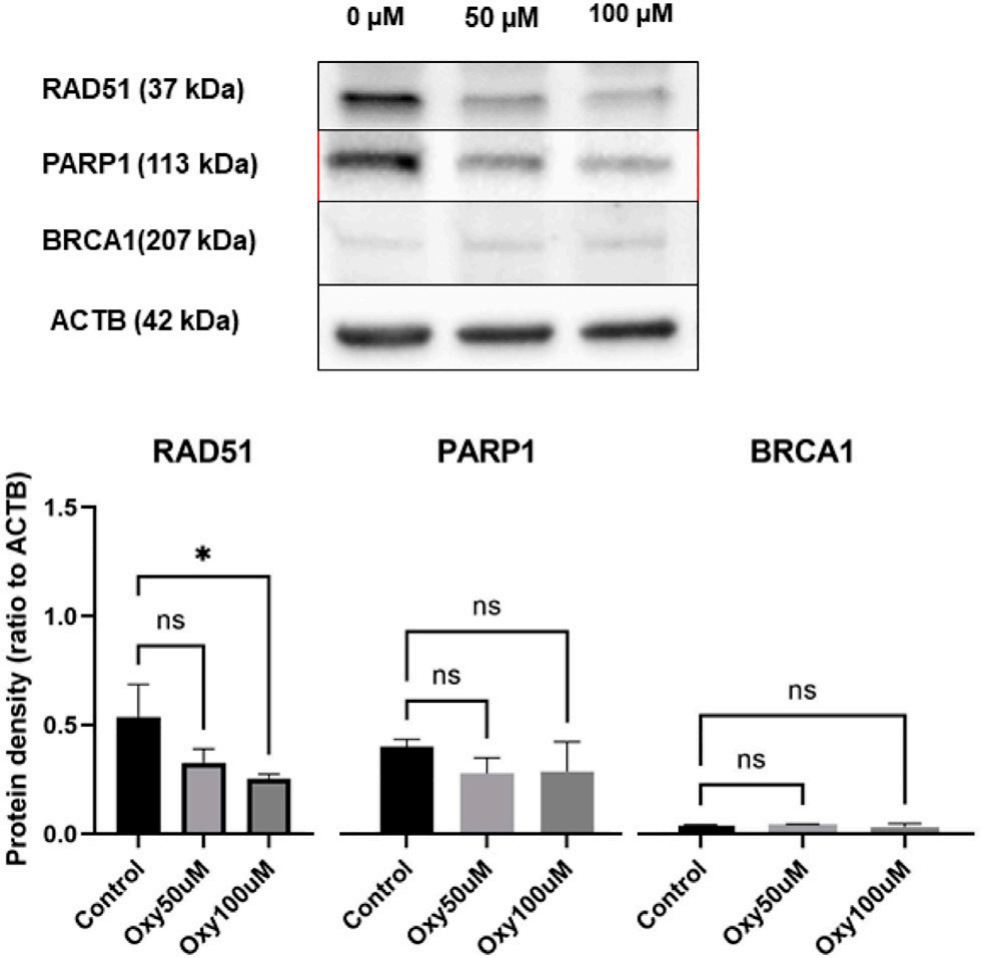
Expression of DNA repair proteins in MCF-7 breast cancer cells treated for 24 h with 50 and 100 µM of OXY compared with untreated control. Total cell lysates were blotted and identified as described in the materials and methods section. Statistical analysis was carried out using One-way ANOVA analysis of variance followed by Dunnett's test. The significant difference was compared relatively to control (*p = 0.0191).

Protein bands of the expected size corresponding to RAD51, PARP1 and BRCA1 in the whole-cell extracts treated with different doses of 50 and 100 µM OXY were observed compared to untreated controls (Figure 11). The protein expression of RAD51 (ratio to ACTB) was significantly different relative to control (0.54 ± 0.15 to 0.33 ± 0.06, and 0.25 ± 0.02), respectively. While PARP1 and BRCA1 expression decreased slightly, but not significantly compared to the control. These proteins were also evaluated for changes in expression in MCF10A cells following treatment with OXY, but expression levels of the proteins of interest were not found to be significantly different from the controls (data not shown).

## Discussion

Microarray-based techniques including GeneChip™ are high-throughput technologies for genetic exploration. These technologies have been developed as a single platform which provides extensive information on gene networks and functions on drug efficacy and toxicity (Taylor et al., 2005). Over the years, this kind of platform has been extensively implemented to evaluate the effect of OXY and derivatives, especially resveratrol on several cancerous cells such as multiple myeloma and colorectal cancer cells (Geng et al., 2018; Lee et al., 2018). However, OXY, in this study is also reported to demonstrate selective toxicity towards MCF-7 cells compared to MCF10A cells and others. The exposure time between the cells and OXY was reduced to 24 h (IC_50_ for MCF-7 cells = 368.37 µM) in microarray and qPCR analysis because the experiment was designed to analyze the gene expression of the main molecular effects at the early stage (Li and Sarkar, 2002). Amongst the OXY-modulated genes observed in MCF-7 cells, the genes that were significantly changed (mostly downregulated) were involved in cell cycle control, DNA replication and DNA synthesis.

Apoptosis is a vital component of cellular processes moderating life and death of cells and therefore is of great therapeutic potential (Kruidering and Evan, 2000). Moreover, Apoptosis, which can cause damage to DNA, is the central mechanism of chemotherapeutics and most plant–derived anticancer drugs (Cragg and Pezzuto, 2016). OXY induced apoptosis through both intrinsic and extrinsic pathways. It is possible in consideration of the results reported here that OXY decreased the expression of anti-apoptotic proteins such as B-cell lymphoma 2 (BCL-2) and increased pro-apoptotic proteins like BAX and BCL-2 -antagonist/killer-1 (BAK). It is then possible that DIABLO and cytochrome c released, activated apoptotic peptidase activating factor 1 (APAF1) forming apoptosomes, which led to the loss of mitochondrial membrane potential. On the other hand, the results reported here suggest that the extrinsic apoptotic pathway may have been initiated by OXY through Fas cell surface death ligand/receptor (FASL/FASR) and tumour necrosis factor-alpha/receptor (TNF-α/TNFR1), activating CASP8, with the subsequent activation of CASP3. Notably, OXY has been previously reported to induce the intrinsic pathway of apoptosis in neuroblastoma cells (Rahman et al., 2017).

CDK2, CDK4 expression plays an important role in G1-S phase checkpoint control normally working together with cyclin E as a complex protein. This complex increases the synthesis of histone proteins and DNA replication (Caruso et al., 2018). Therefore, the G0/G1 and S phases of cell cycle were arrested. CDK2 is also inhibited by CDKN1A, which represents a major target of p53 activity. Interestingly, TP53 remained unchanged in response to the treatment, which was consistent to the previous study reported that OXY moderated p53-independent S phase arrest, ROS–independent apoptosis in neuroblastoma cells (Rahman et al., 2017). The results here illustrate that OXY induced apoptosis in the cells but did not activate TP53, which was responsible for the DNA damage. However, p53-independent upregulation of CDKN1A may be induced by some genes in cell cycle, apoptosis or senescence and autophagy in cancer pathways (Aliouat-Denis et al., 2005). Recently, CDK inhibitors have been investigated as a possible chemotherapeutic (Wood et al., 2019). OXY significantly decreased the expression of CDK4 and E2F1, which subsequently conducted cell cycle arrest.

OXY-treated MCF-7 cells induced the expression of autophagic genes including microtubule-associated protein one light chain 3 beta (*MAP1LC3B*), sequestosome 1 (*SQSTM1*) and GABA(A) receptor-associated protein like 2 (*GABARAPL2*). Autophagy or self-eating is an evolutionarily conserved catabolic pathway in eukaryotes playing a significant role in the recycling of cellular components as part of a housekeeping function with Beclin1 and Atg5 reported to play important roles (Bilir et al., 2002). Autophagy-mediated cell death can be the starting process of apoptosis, and blocking caspase activity can cause a cell to enter autophagy cell death instead of apoptosis (Lockshin and Zakeri, 2004). OXY has been previously reported to be capable of inducing neuroblastoma cell death by autophagy *via* the PI3K/AKT/mTOR pathway (Rahman et al., 2017).

OXY dose-dependently mediated down regulation of DNA repair gene expression (PARP1, RAD51 and BRCA1) in MCF-7 cells. The significantly down-regulated expression of RAD51 by OXY may be the key mechanism by which OXY mediates its anticancer effect in consideration of RAD51 being overexpressed in a variety of cancer cells (Raderschall et al., 2002; Hannay et al., 2007; Klein, 2008). The principle of inhibiting DNA repair genes has been proposed for cells that carry mutations in the breast cancer susceptibility genes *BRCA1* or *BRCA2* as a possible cancer drug target (Farmer et al., 2005). Previously Walsh (2015) reported that a deficiency in *PARP* or *BRCA* alone had no impact on cancer cell viability, but a deficiency in both leads to a lethal effect on cancer cells. The western blot analysis presented here demonstrates that OXY inhibited the protein expression of RAD51 and slightly decreased the expression of PARP1 and BRCA1. Moreover, the inhibition of RAD51 also enhanced cytotoxicity and apoptosis induction in cancer stem cells (Ruiz et al., 2018) Oxyresveratrol’s property of apoptosis activation, cell cycle arrest, cell senescence and autophagy including the implication of inhibiting RAD51 may be able to facilitate an increase in the efficacy of commonly used chemotherapeutics.

## Conclusion

In this research report, we clearly demonstrate that OXY regulates the expression of various genes, which have important roles in carcinogenesis-associated pathways. OXY enhanced the apoptosis cascade through both extrinsic and intrinsic pathways. OXY also decreased the expression of *CDK2*, *CDK4* and *E2F1*, affecting the initial stage of the cell cycle (G1-S phase). More interestingly, the compound induced a down-regulation of genes and protein activities involved in DNA repair, including RAD51 and PARP1. These results indicate that OXY may be utilized to overcome drug resistance and enhance the efficacy of chemotherapy drugs.

## Data Availability

The original contributions presented in the study are publicly available. This data can be found here: https://www.ncbi.nlm.nih.gov/, GSE151139.
